# Immune cell phenotype and function patterns across the life course in individuals from rural Uganda

**DOI:** 10.3389/fimmu.2024.1356635

**Published:** 2024-03-18

**Authors:** Angela Nalwoga, Marjorie Nakibuule, Romin Roshan, Moses Kwizera Mbonye, Wendell Miley, Denise Whitby, Robert Newton, Rosemary Rochford, Stephen Cose

**Affiliations:** ^1^ Department of Immunology and Microbiology, University of Colorado, Aurora, CO, United States; ^2^ Medical Research Council/ Uganda Virus Research Institute and London School of Hygiene & Tropical Medicine, Entebbe, Uganda; ^3^ Frederick National Laboratory for Cancer Research, Viral Oncology Section, AIDS and Cancer Virus Program, Leidos Biomedical Research, Inc., Frederick, MD, United States; ^4^ Department of Health Sciences, University of York, York, United Kingdom; ^5^ Department of Clinical Research, London School of Hygiene & Tropical Medicine, London, United Kingdom

**Keywords:** immune parameters, immune phenotypes, Epstein-Barr virus T cell responses, Uganda, lifecourse

## Abstract

**Background:**

To determine the pattern of immune cell subsets across the life span in rural sub-Saharan Africa (SSA), and to set a reference standard for cell subsets amongst Africans, we characterised the major immune cell subsets in peripheral blood including T cells, B cells, monocytes, NK cells, neutrophils and eosinophils, in individuals aged 3 to 89 years from Uganda.

**Methods:**

Immune phenotypes were measured using both conventional flow cytometry in 72 individuals, and full spectrum flow cytometry in 80 individuals. Epstein-Barr virus (EBV) IFN-γ T cell responses were quantified in 332 individuals using an ELISpot assay. Full blood counts of all study participants were also obtained.

**Results:**

The percentages of central memory (T_CM_) and senescent CD4+ and CD8+ T cell subsets, effector memory (T_EM_) CD8+ T cells and neutrophils increased with increasing age. On the other hand, the percentages of naïve T (T_N_) and B (B_N_) cells, atypical B cells (B_A_), total lymphocytes, eosinophils and basophils decreased with increasing age. There was no change in CD4+ or CD8+ T effector memory RA (T_EMRA_) cells, exhausted T cells, NK cells and monocytes with age. Higher eosinophil and basophil percentages were observed in males compared to females. T cell function as measured by IFN-γ responses to EBV increased with increasing age, peaking at 31-55 years.

**Conclusion:**

The percentages of cell subsets differ between individuals from SSA compared to those elsewhere, perhaps reflecting a different antigenic milieu. These results serve as a reference for normal values in this population.

## Introduction

Chronic herpesvirus infections are common across the globe (1, 2); 90% of the adult human population worldwide is infected with Epstein-Barr virus (EBV) (3) and 83% are infected with cytomegalovirus (CMV) (4). However, in sub-Saharan Africa (SSA), the prevalence of herpesvirus infections is higher than elsewhere, and primary infections occur early during childhood. By age five years, over 90% of children in SSA are seropositive for HSV-1, CMV and EBV (5–8) compared to <50% in high-income countries (9–11). Early infection with chronic viruses has implications for increased risk for the diseases associated with these viruses (6). Similarly, acute, repeated infections, such as *Plasmodium falciparum* malaria are also common in SSA (12). Differences in the antigenic milieu in SSA may impact the immune profiles of individuals, compared to other settings (13). Such data, however, are scarce, at least for SSA.

Immunosenescence and immune exhaustion play a role in disease severity and susceptibility globally (14–16). Immunosenescence is characterised by shortened telomeres, reduced telomerase activity, a reduced frequency of naive T cells and reduced cellular proliferative ability (17, 18), and an increase in terminally differentiated T cells. In addition to ageing, chronic viral infections such as HIV, CMV, EBV and hepatitis B viruses have been shown to drive premature senescence in young individuals (19). T cell exhaustion is characterised by high expression of inhibitory molecules on cell surfaces such as PD1, TIGIT, LAG3, TIM3 and CTLA4, low proliferative capacity and impaired effector functions (cytokine production and cytotoxicity) (20). It has been hypothesised that, in SSA, where both chronic and acute infections are both widespread and frequent, both early onset immunosenescence and T cell exhaustion may be more common (21).

Using conventional flow cytometry, single-cell analysis of immune markers has been limited to up to 18 cellular markers due to spectral overlap of the fluorophores (22). As a consequence, immunophenotype analysis of human clinical samples typically focuses on single lymphocyte subsets (*e.g.* evaluation of CD4^+^ T cell subsets or CD19^+^ B cell subsets). The advent of full spectrum flow cytometry addresses this challenge by using differences in full emission spectra signatures across all lasers, allowing much larger fluorescent panels (>40 antibodies) to be used in a single analysis (23). We characterised the major cell types in peripheral blood, including T cells, B cells, monocytes and NK cells using both conventional and full-spectrum flow cytometry.

## Methods

### Study design

In 2017, we nested a cross-sectional study of 975 individuals within the rural Ugandan General Population Cohort (GPC), investigating the determinants of Kaposi’s sarcoma-associated herpesvirus transmission (24). The GPC is a rural community-based cohort of about 22,000 people in 25 adjacent villages in southwestern Uganda (25, 26). After stratification for age and sex, HIV-negative, healthy individuals (without reported illnesses) aged 3 to 89 years were randomly selected for enrolment in this cross-sectional study. Blood was collected in both ACD and EDTA tubes, and demographic data were recorded using questionnaires. Peripheral blood mononuclear cells (PBMCs) were isolated from whole blood using density gradient centrifugation within two hours of sample collection. Viable PBMCs in freezing media (10% DMSO, 90% FBS) were stored in liquid nitrogen. Plasma from ACD tubes was stored at minus 80°C.

### Ethical approvals

The study was approved by the Uganda Virus Research Institute Research and Ethics Committee (UVRI-REC, reference number: GC/127/16/09/566), the Uganda National Council for Science and Technology (UNCST, reference number: HS2123) and the London School of Hygiene and Tropical Medicine Ethics Committee (reference number: 11881). Written informed consent was obtained from all adults aged 18 years and above. Children below 18 years consented to the study via a parent or guardian; we also sought, in addition to parental consent, written assent from children aged between 8 and 17 years.

### Study participants selection and laboratory analysis

A full blood count was performed on 975 individuals (24) but only 697 individuals aged 3 to 89 (mean age of 37) were included in this manuscript. This was because we wanted to include only healthy individuals; those with parasitic infections (*Plasmodium falciparum* malaria and helminths) and incomplete health data were excluded. Study participants were analysed for immune phenotypes using both conventional flow cytometry and full-spectrum flow cytometry. Conventional flow cytometry was undertaken at the Uganda Virus Research Institute (UVRI) before gaining access to the more advanced full-spectrum flow cytometer (5 laser Aurora Cytek) at the University of Colorado. Due to variability between instruments, data from the two machines were not directly compared or added together. Using data acquired from conventional flow cytometry, cell subsets were compared across four age groups (4-15, 16-30, 31-55 and 56-89). Using data acquired from full spectrum flow cytometry, cell subsets were compared across three age groups (16-30, 31-55 and 56-89). In addition, conventional flow cytometry analysis included children, whereas full spectrum flow cytometry did not.

### Laboratory analysis

#### Full blood *count and multiplex bead assay*

Blood in EDTA tubes was analysed for immune cell parameters using the Ac.T 5 diff CP haematology analyser (Beckman Coulter) following the manufacturer’s instructions. IgG antibody levels to the EBV viral capsid protein VCA were measured in plasma using a multiplex bead assay on a Luminex BioRad Bio-plex200 system as previously reported (27).

#### Enzyme-linked immunosorbent Spot (ELISpot) assay

IFN-γ T cell responses to a cocktail of latent and lytic EBV peptides (**Supplementary Table 1**) were measured using the ELISpot assay. The MABTECH Human IFN-γ ELISpot kit (Code: 3420-2AST-2) was used for the assay, with a few alterations to the manufacturer’s protocol. Briefly, the ELISpot plates with the capture antibody from the kit were washed five times with 200μl of 1xPBS per well. Afterwards, thawed cells were added to the plates in a volume of 100μl AIM-V medium containing 150,000 cells per well. The plates were covered with the lid, wrapped in aluminium foil and transferred to a 5% CO_2_ 37°C incubator for a 24-hour resting period. To stimulate them, 100μl per well of the EBV peptide pool, anti-CD3 and media (AIM-V media, Gibco 12055091) at working concentrations of 5μg/ml/peptide were added to the wells. The plates were then incubated at 5% CO_2_ 37°C for a further 46-48 hours. Following stimulation, cells were washed 5 times with 200μl of PBS per well and 100μl of anti-human IFN-γ IgG conjugated to alkaline phosphatase (Code: 7-B6-ALP) was added at a dilution of 1/200 in PBS + 0.5% FBS. The plates were incubated at room temperature (25°C) for 2 hours. After the incubation, the plates were washed 5 times with 200μl of 1xPBS per well and 100μl of filtered 5-bromo-4-chromo-3-indolyl-phosphate (BCIP)/nitroblue tetrazolium (NBT)-plus substrate were added per well. The plates were then incubated at room temperature for 6.5 minutes and the reaction was stopped by washing the plate with running tap water. The plates were dried in the dark overnight and the spots were subsequently counted using an ELISpot reader (CTL ImmunoSpot Analyzer). This protocol has been reported elsewhere (28, 29).

#### Flow cytometry

Fluorochrome antibody conjugate titration and reference control type selection were carried out prior to study participants’ PBMCs staining (**Table 1**). For conventional flow cytometry, beads (BD CompBeads (BD Biosciences, 552843) were used for compensation of all fluorochrome antibody conjugates apart from the live/dead stain where PBMCs were used (**Table 1**). The most appropriate reference control type beads (Ultra Comp eBeads Invitrogen, 01-2222-42) or PBMCs were used for full spectrum flow cytometry (**Table 1**). Live/dead staining using the fixable viability dye eFluor 780 (eBioscience) for conventional flow cytometry, or fixable blue dead stain kit for full spectrum flow cytometry (Thermo Fisher) was carried out in 1mL of PBS containing 1 million PBMCs. IgG F_C_ receptor (F_C_R) blocking was performed prior to fluorochrome antibody conjugate staining using a human F_C_R binding inhibitor (eBioscience). Fluorochrome antibody conjugate cocktails were made in FACS buffer (1X PBS, 0.5% BSA, 0.5M EDTA and 0.05% sodium-azide) using the predetermined optimal concentration (**Table 1**). Brilliant stain buffer (BD Biosciences 566349) was added to the antibody cocktail following the manufacturer’s recommendations. Study participants’ PBMCs were stained with a cocktail of fluorochrome antibody conjugates in 100ul or 50ul of FACS buffer for full spectrum or conventional flow cytometry, respectively, for 30 minutes at 4^°^C. Stained PBMCs were fixed using the FluoroFix buffer (BioLegend, 422101) before acquisition on BD LSR-II flow cytometer (conventional flow cytometer) or a 5-laser Cytek Aurora (full spectrum flow cytometer). A total of 200,000 events from each study participant sample were recorded.

**Table 1:** Specifications of antibodies used in flow cytometry.

Antibody	Fluorochrome	Clone	Volume (mL)	Reference control	Catalogue number	Vendor
Full spectrum flow cytometry						
CD4	Brilliant Violet 510	OKT4	2.5	Beads*	317443	BioLegend
CD57	PE	HNK-1	5	PBMCs**	359611	BioLegend
CD16	Brilliant Violet 785	3G8	2.5	Beads*	302045	BioLegend
CD38	Brilliant Violet 421	HIT2	2.5	Beads*	303525	BioLegend
KLRG1	PE/Cy7	SA231A2	1	Beads*	367719	BioLegend
CD223 (LAG-3)	APC/Fire 750	11C3C65	5	Beads*	369329	BioLegend
CD279 (PD-1)	Brilliant Violate 711	EH12.2H7	5	Beads*	329927	BioLegend
HLADR	PE/cy5	L243	2	Beads*	307607	BioLegend
CCR7	Brilliant Violet 750	G043H7	2.5	Beads*	353253	BioLegend
CD45RA	Brilliant Violet 650	HI100	2.5	Beads*	304136	BioLegend
CD21	PE/Dazzle 594	Bu32	0.625	Beads*	354921	BioLegend
IgD	Alexa fluor 700	1A6-2	0.625	Beads*	348229	BioLegend
CD10	PerCp-Cy5.5	HI10a	5	Beads*	312215	BioLegend
CD8	BUV 805	SK1	2.5	PBMCs	612889	BD Biosciences
CD19	BUV 395	SJ25C1	1.25	PBMCs	563551	BD Biosciences
CD3	Alexa fluor532	UCHT1	5	PBMCs	58-0038-41	ThermoFisher scientific
CD28	Brilliant Violet 605	CD28.2	5	PBMCs	302967	BioLegend
CD27	APC	O323	2	PBMCs	302809	BioLegend
CD56	BUV 737	NCAM16.2	0.5	PBMCs	564448	BD Biosciences
CD96	BB515	6F9	5	Beads*	564774	BD Biosciences
CD314 (NKG2D)	Alexa Fluor 660		1	Beads*	320841	BioLegend
CD14	Brilliant Violet 480	M5E2	5	Beads*	746304	BD Biosciences
NKG2A (CD159a)	BUV 615	131411	5	Beads*	752302	BD Biosciences
fixable blue dead cell stain kit					L34961	Thermo Fisher Scientific
Conventional flow cytometry						
CD5	PE	UCHT2	0.5	Beads***	555353	BD Biosciences
IgM	PE-Cy5	G20-127	1	Beads***	551079	BD Biosciences
CD38	PE/Cy7	HB-7	0.5	Beads***	356608	BioLegend
IgD	PE-CF594	IA6-2	0.25	Beads***	562540	BD Biosciences
IgG	Alexa Fluor 700	G18-145	2.5	Beads***	561296	BD Biosciences
CD27	Brilliant Violet 421	O323	0.25	Beads***	302824	BioLegend
CD28	Brilliant Violet 421	CD28.2	1	Beads***	562613	BD Biosciences
CD57	PE	NK-1	0.03	Beads***	560844	BD Biosciences
HLA-DR	PE-Cy7	G46-6	0.25	Beads***	560651	BD Biosciences
CD4	PE/Cy5	RPA-T4	0.03	Beads***	300510	BioLegend
CD279 (PD-1)	FITC	EH12.2H7	2.5	Beads***	329904	BioLegend
CD197 (CCR7)	PE-CF594	150503	0.5	Beads***	562381	BD Biosciences
CD8a	Brilliant Violet 570	RPA-T8	1	Beads***	301038	BioLegend
CD3	Brilliant Violet 650	5K7	1	Beads***	563999	BD Biosciences
CD38	Brilliant Violet 421	HIT2	0.25	Beads***	562444	BD Biosciences
CD10	Brilliant Violet 650	HI10a	2.5	Beads***	563734	BD Biosciences
CD21	FITC	Bu32	0.03	Beads***	354910	BioLegend
CD19	APC	SJ25C1	0.125	Beads***	345791	BD Biosciences
IgG	Alexa Flour700	G18-145	2.5	Beads***	561296	BD Biosciences
CD45RA	APC	HI100	1	Beads***	550855	BD Biosciences
Fixable viability dye	eFlour 780		NA	PBMCs**	65-0865-18	eBioscience

*Ultra Comp eBeads (Invitrogen, Catalogue number: 01-2222-42); **PBMCs, peripheral blood mononuclear cells, ***BD CompBeads (Catalogue number: 552843).

#### Conventional flow cytometry

Three different panels were used to identify T and B cells using conventional flow cytometry. Panel one contained CD3, CD4, CD8, CCR7, CD45RA and live/dead, panel two contained CD3, CD4, CD8, CD57, CD28, HLADR, PD-1 and live/dead. Panel three contained CD19, CD10, CD5, CD27, IgD, CD21, CD38, IgM, IgG and the live/dead stain. Single cells were gated using forward scatter area and forward scatter height. Lymphocytes were gated using side scatter and forward scatter followed by the exclusion of dead cells using the live/dead stain (**Supplementary Figure 1** and **Supplementary Table 1**).

#### Full spectrum flow cytometry

**Figure 1:**
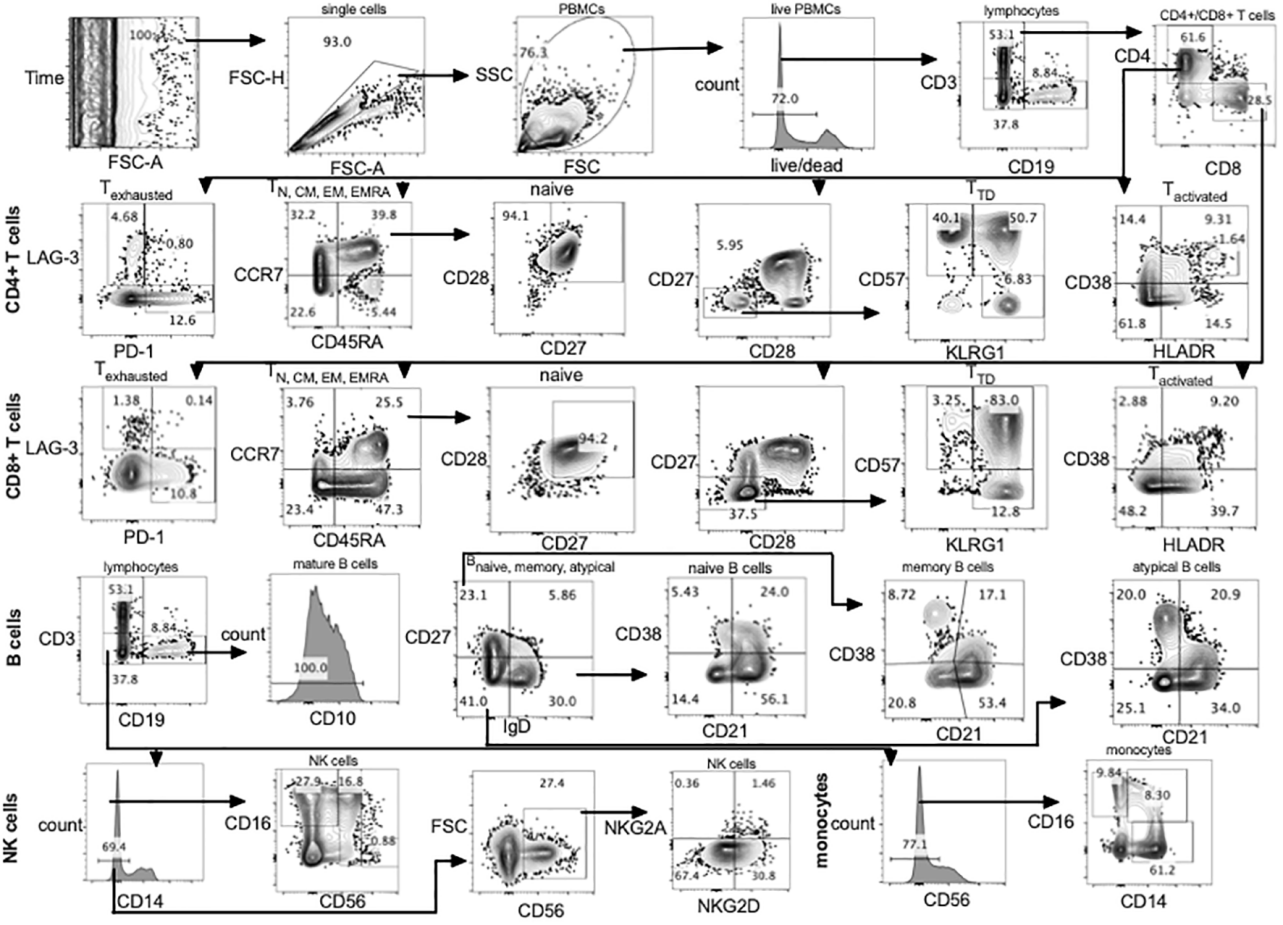
Gating strategy using full spectrum flow cytometry. CD4+, CD8+ T cells, B cells, NK cells and monocytes were gated using flowJo 10.8.1 software following acquisition on a 5 laser Cytek Aurora cytometer. TD, terminally differentiated; N, naïve; CM, central memory; EM, effector memory; TEMRA, terminally differentiated effector memory; PBMCs, peripheral blood mononuclear cells.

Using a single panel of 23 antibody-fluorochrome conjugates and one live/dead stain (fixable blue dead cell stain kit (Thermo Fisher, L34961) T cell, B cell, NK cell and monocyte subsets were identified (**Figure 1**; **Supplementary Table 1**). Single cells were gated using forward scatter area and forward scatter height. Lymphocytes and monocytes were gated using side scatter and forward scatter followed by exclusion of dead cells using the live/dead stain (**Figure 1**). Using CD3 and CD19 three main subsets were classified including CD3+ (T cells) CD19+ (B cells) and CD19-CD3- (NK cells and monocytes).

#### EBV real-time PCR

EBV DNA was quantified in PBMCs and saliva using primers (Balf5 EBV forward: 5’ – CGG AAG CCC TCT GGA CTT C – 3’, - Balf5 EBV reverse: 5’ – CCC TGT TTA TCC GAT GGA ATG – 3’) and probe (Balf5 EBV Probe: 5’ -/56-FAM/TGT ACA CGC ACG AGA AAT GCG CCT/3BHQ_1/- 3’) previously reported to be specific to the Balf5 gene (6, 30). Additionally, B-Actin was amplified in the same sample as an internal positive control using primers (B-Actin forward: 5’ – TCA CCC ACA CTG TGC CCA TCT ACG A – 3’, B-Actin reverse: 5’ – CAG CGG AAC CGC TCA TTG CCA ATG G – 3’) and probe (B-Actin Probe: 5’ -/5HEX/ATG CCC TCC CCC ATG CCA TCC TGC GT/3BHQ_1/- 3’) as previously reported (31).

### Statistical analysis

Flow cytometry data were acquired on an LSR-II (for conventional flow cytometry) and Cytek Aurora (for full spectrum flow cytometry) and analysed using FlowJo software version 10.8.1. Statistical analysis was performed using STATA version 13 (StataCorp, College Station, Texas USA) and GraphPad Prism version 8.0.1 for graphical representation. Both nonparametric tests including Mann-Whitney and Kruskal Wallis, Spearman’s rank correlation as well as parametric tests including one-way ANOVA and student T-test were used for statistical analysis of quantitative data appropriately. False Discovery Rate (FDR) was used to adjust for multiple comparisons. Logistic regression analysis adjusting for testing batch and sex as well as the chi^2^ test were used to analyse qualitative IFN-γ responses to EBV by age groups.

## Results

A total of 72 individuals aged 4 to 88 years with a mean age of 36 years were tested for immune phenotypes using conventional flow cytometry. Eighty individuals aged 16 to 89 years with a mean age of 45 years were tested for immune phenotypes using full spectrum flow cytometry. These same 80 individuals were tested for EBV IFN-γ T cell responses. Additional individuals were tested for IFN-γ responses to EBV, bringing the total to 332 individuals aged 3 to 89, with a mean age of 34 years, tested for EBV IFN-γ T cell responses. More details of the characteristics of the participants selected for all the analyses are shown in **Table 2**.

**Table 2:** Study population characteristics.

	Full spectrum flow cytometry	Convectional flow cytometry	Full blood count	EBV* ELISPOT**
Age, median (IQR)-years	46 (26-64) N=80	35 (14-55) N=72	35 (18-52) N=697	32 (16-50) N=332
Age groups-years percentages 3-15 16-30 31-55 56-89	31% (25/80) 38% (30/80) 31% (25/80)	31% (22/72) 17% (12/72) 30% (21/72) 24% (17/72)	21% (144/697) 23% (157/697) 36% (254/697) 20% (142/697)	23% (76/332) 26% (87/332) 27% (106/332) 24% (63/332)
Age groups-years median (IQR) 3-15 16-30 31-55 56-89	22 (18-24) N=25 46 (35-51) N=30 69 (66-74) N=25	10 (5-13) N=22 23 (21-26) N=12 48 (40-53) N=21 68 (61-71) N=17	9 (7-13) N=144 23 (18-27) N=157 43 (37-50) N=254 67 (62-72) N=142	7 (6-10) N=76 22 (18-26) N=87 43 (27-48) N=106 69 (65-73) N=63
Sex, males	50% (40/80)	44% (32/72)	49% (336/691)	52% (171/332)

*EBV, Epstein-Barr virus, **ELISpot, Enzyme linked immunosorbent spot, IQR, Interquartile range.

### CD4+ and CD8+ T cell subsets by age

#### Naïve, central memory, effector memory and terminally differentiated T cells

**Figure 2:**
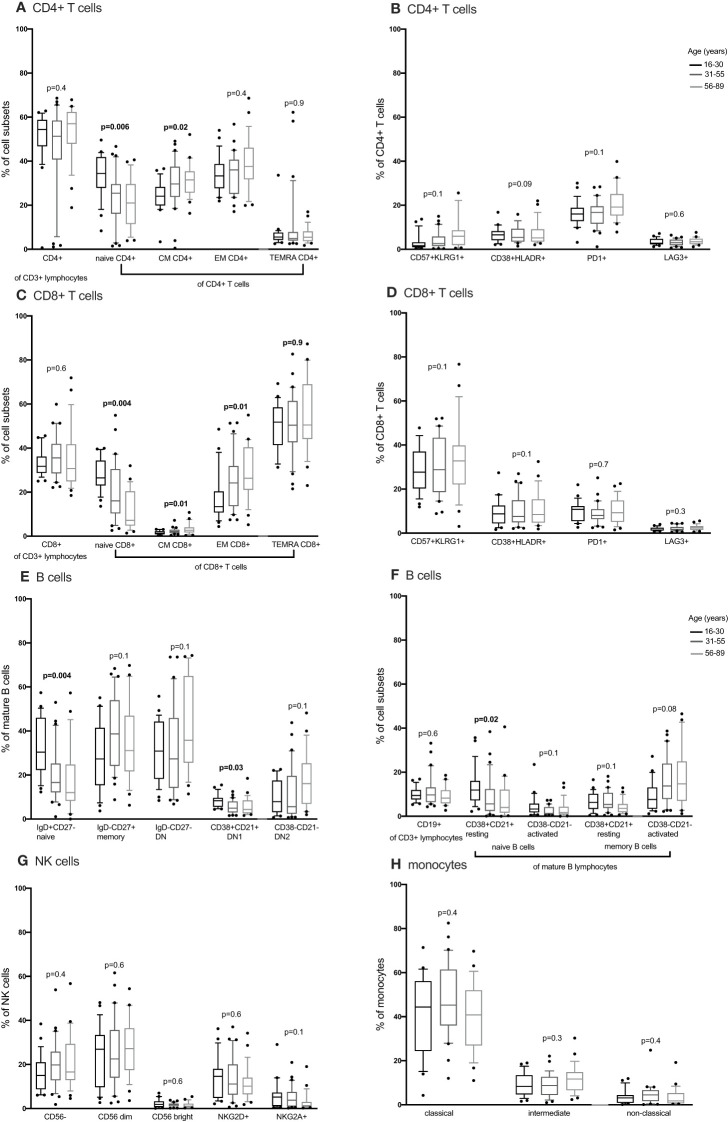
The distribution of CD4+ T cells, CD8+ T cell, B cells, NK cells and monocytes subsets in individuals aged 16 to 89 years (16-30, N= 25 31-55, N= 30 56-89, N= 25) using full spectrum flow cytometry. CD4+ T cells, CD8+ T cell, B cells, NK cells and monocytes were gated using flowJo 10.8.1 software following acquisition on a 5 laser Cytek Aurora cytometer. DN, double negative; CM, central memory; EM, effector memory; TEMRA, terminally differentiated effector memory. P values obtained from a Kruskal Wallis test. False discovery rate (FRD) used to adjust for multiple comparisons. Parent population is shown in Y-axis label **(B, D, E, G, H)** or below the subset label **(A, C, F)**.

**Figure 3:**
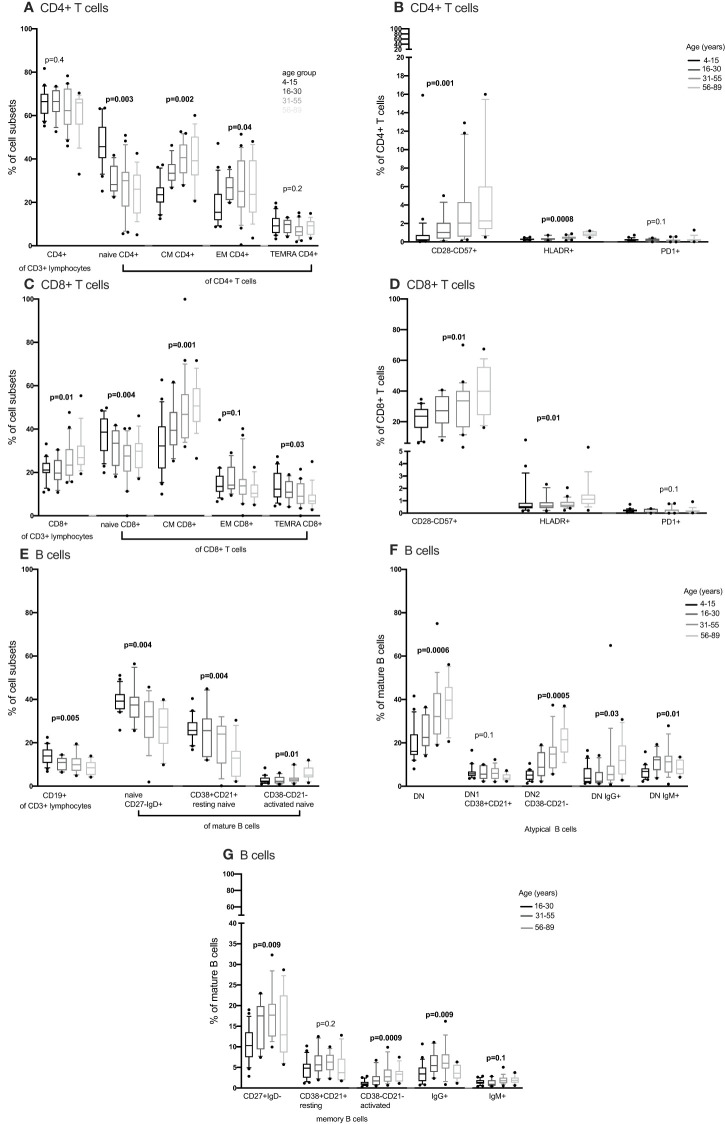
The distribution of CD4+ T cells, CD8+ T cell and B cells subsets in individuals aged 4 to 89 years (4-15, N=22 16-30, N= 12 31-55, N= 21 56-89, N= 17) using convetional flow cytometry. CD4+ T cells, CD8+ T cell and B cells were gated using flowJo 10.8.1 software following acquisition on an LSR-II flow cytometer using three antibody panels. DN, double negative; CM, central memory; EM, effector memory; TEMRA, T effector memory RA. P values obtained from a Kruskal Wallis test. False discovery rate (FRD) used to adjust for multiple comparisons. Parent population is shown in Y-axis label **(B, D, F, G)** or below the subset label **(A, C, E)**.

**Figure 4:**
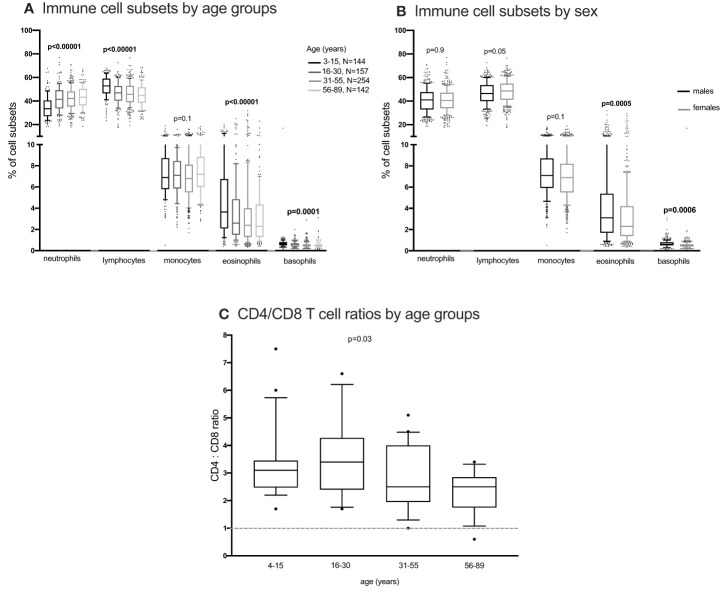
The distribution of immune cells subsets in whole blood. EDTA whole blood was analysed for differential immune cell subsets using the FACScalibur. Percent cell subsets were compared between age groups and sex using one-way ANOVA **(A, C)** and student T test **(B)** respectively.

We compared CD4+ and CD8+ T cell subsets in the 4-15, 16-30, 31-55 and 56-89 age groups using the Kruskal Wallis test. Overall, the percentage of naïve CD4+ and CD8+ T cells decreased with increasing age groups (**Figures 2A, C**, **3A, C**; **Supplementary Figures 2**-**5**, **Supplementary Tables 2**, **3**). The median of naïve CD4+ T cells was 46% interquartile range-IQR (41-54) of total CD4+ T cells in the 4-15 age group, 28% IQR (25-37) in the 16-30 age group, 30% IQR (18-34) in the 31-55 age group and 26% IQR (15- 32) in the 56-89 age group (**Figure 3A**; **Supplementary Table 3**). The median percentage of naïve CD8+ T cells of total CD8+ T cells was 39% IQR (31-45) in the 4-15 age group, 34% IQR (23-38) in the 16-30 age group, 28% IQR (22- 32) in the 31-55 age group and 30% IQR (24-31) in the 56-89 age group (**Figure 3C**). Overall, percentages of central memory CD4+ and CD8+ T cells increased with increasing age groups (**Figures 2A, C**, **3A, C**). Individuals aged 4-15 years had a lower percentage of effector memory CD4+ T cells compared to their older counterparts (**Figure 3A**). The percentage of effector memory CD4+ T cells didn’t change between 16 to 89 years (**Figures 2A**, **3A**). When age was analysed continuously, the percentages of effector memory CD4+ T cells slightly increased with increasing age (**Supplementary Figures 2**, **3**). The percentage of effector memory CD8+ T cells increased with increasing age groups (**Figure 2C**). The percentage of total CD4+ T cells, total CD8+ T cells and T_EMRA_ didn’t change by age group (**Figures 2A, C**, **3A, C**). However CD4+ T cells, CD8+ T cells and T_EMRA_ had a weak positive correlation with increasing age (**Supplementary Figures 2**-**5**). However, the ratio of CD4+:CD8+ T cells was highest in the 16-30 age groups, and lowest in the 31-89 groups (**Figure 4C**). Direct comparison between conventional flow cytometry and full spectrum flow cytometry was not possible with the current data, however, CD4+ naïve CD4+, effector memory CD4+, T_EMRA_ CD4+, naïve CD8+, central memory CD8+, T_EMRA_ CD8+ T cells followed a similar pattern in both results from the two flow cytometry methods. On the other hand, the pattern of central memory CD4+, CD8+, effector memory CD8+ by full spectrum flow cytometry was different from the pattern of the same cell subsets by conventional flow cytometry (**Figures 2**, **3**; **Supplementary Tables 2**, **3**). We were not equipped to investigate the differences in cell sub sets by the two flow cytometry methods due to sample limitations but these differences could be partially attributed to the inclusion of the 4-15 years age group in conventional flow cytometry analysis but not the full spectrum flow cytometry.

#### Activated, exhausted and senescent T cells

We next obtained the percentages of CD4+ and CD8+ senescent (CD57+ CD28-), activated (HLADR+CD38+) and exhausted (PD1+ or LAG3+) subsets from the total CD4+ and CD8+ T cells respectively. Whilst Follicular Helper T cells do express PD1, they don’t express LAG3. Whilst we cannot rule out TFH cells, we can be reasonably sure that LAG3 expressing cells are not this subset and are likely exhausted. Although percentages of senescent CD8+ T cells were higher than the percentages of senescent CD4+ T cells [median 27% IQR (18.6 – 39.7) vs. 1% IQR (0.37 – 2.85)], both senescent CD8+ and CD4+ T cells increased with increasing age (**Figures 3B, D**). The percentages of activated (HLADR+) CD4+ and CD8+ T cells were low and increased with increasing age groups (**Figures 3B, D**). The percentages of exhausted T cells were very low and did not change with increasing age (**Figures 2B, D**, **3B, D**).

#### B cells

Concurrently, we obtained the percentages of B cell subsets out of the total CD19+ B cells and compared them the 4-15, 16-30, 31-55 and 56-89 age groups using the Kruskal Wallis test. The percentages of total CD19+ B cells decreased with increasing age groups (**Figure 3E**). Naïve B cells also reduced with increasing age groups (**Figures 2E**, **3E**). Resting naïve B cells were relatively high in individuals aged 4-55 and lower in 56-89 year-olds (**Figure 3E**). Activated naïve B cells increased with age (**Figure 3E**). Atypical B cells are mature B cells double negative for both CD27 and IgD. These double negative (DN) mature B cells were classified into DN1 and DN2 using CD38 and CD21 (32). Atypical B cells, DN2 and IgG+ double negative B cells increased with increasing age groups (**Figure 3F**). DN1 B cells decreased with increasing age groups (**Figure 2F**). DN IgM+ B cells increased with increasing age groups and started reducing in the 31-55 age group (**Figure 3F**). Memory B cells increased with increasing age groups and dropped in the 56-89 age group (**Figure 3G**). Activated memory B cells increased with increasing age groups while IgG+ memory B cells increased with increasing age groups and reduced in the 56-89 age group (**Figure 3G**). Other B cell subsets did not change over time. The pattern of naïve B cells, CD38+ CD21+ naïve B cells with increasing age, measured using full spectrum flow cytometry were similar to the pattern of the same B cell subsets measured using conventional flow cytometry. However, the pattern of CD19+ CD38- CD21- naïve B cells, DN, DN1, DN2 measured using full spectrum flow cytometry were different to the pattern of the same B cell subsets measured using conventional flow cytometry (**Figures 2**, **3**; **Supplementary Figures 6**-**8**).

#### Differences by sex and other cell types

Using the Kruskal Wallis test, NK cells and monocyte subsets did not change with changing age (**Figures 2G, H**; **Supplementary Figure 9**). Neutrophils and lymphocytes obtained from full blood counts were the most abundant and basophils were the least abundant (**Figure 4**). Using the one-way ANOVA, neutrophils increased while lymphocytes decreased with age, both plateauing in the 16-30 age group (**Figure 4A**). Both eosinophils and basophils from full blood counts decreased with increasing age groups, plateauing at the 6-30 year age group, while monocytes did not change in the different age groups. Although the different T, B, NK cells, neutrophils and monocyte subsets were not different between males and females (**Supplementary Figures 10**, **11**) using a student T test, eosinophil and basophil percentages were higher in males compared to females (**Figure 4B**).

#### T cells, antibody responses to EBV and EBV viral load

**Figure 5:**
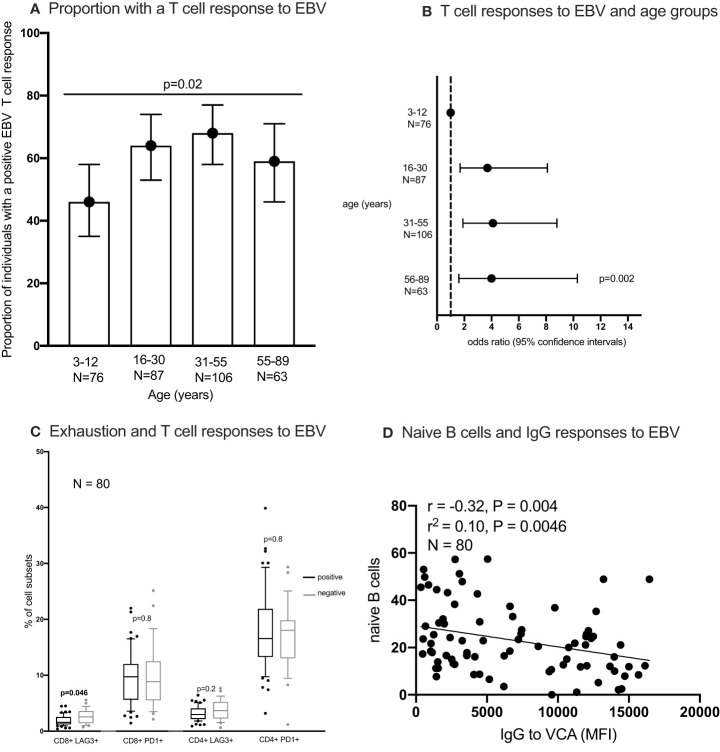
The proportion of individuals with a T cell response to EBV **(A, B)** the percentages of exhausted T cells in individuals with and without an IFN-γ responses to EBV **(C)** and correlation between naïve B cells and IgG responses to EBV **(D)**. IFN-γ responses to EBV (Epstein-Barr virus) peptide pool were measured using enzyme linked immunosorbent spot (ELISpot) assay; IgG to EBV-VCA (viral capsid antigen) was quantified using multiplex bead assay. Statistical analysis methods used include chi^2^ test **(A)**, logistics regression **(B)**, Wilcoxon Rank Sum Test **(C)**, Spearman’s rank correlation **(D)**. naïve B cells were classified as IgD+ CD27- CD10- CD19+ cells.

All individuals tested had antibodies to the EBV VCA antigen, implying that all were infected with EBV. Using the Chi^2^ test, the percentage of individuals with a positive T cell response to EBV was highest in individuals aged 31-55 years and lowest in the youngest age group (3-12 years) (**Figure 5A**). After adjusting for sex and testing batch using logistic regression modelling, individuals in the older age groups were more likely to have a positive EBV T cell response compared to the youngest age group (3-12 years) (**Figure 5B**). Nonetheless, using the Wilcoxon Ranksum test individuals with a positive EBV T cell response had lower percentages of exhausted CD8+ T cells (LAG3+) compared to those without a detectable EBV T cell response (**Figure 5C**). No difference in other cell types were observed between individuals with and without an EBV T cell response (**Figure 5C**; **Supplementary Figure 12C**). Furthermore, the proportion of naïve B cells but not memory or atypical B cells (**Supplementary Figures 12A, B**) negatively correlated (Sperman’s rank correlation) with the amount of IgG to EBV-VCA antigen (**Figure 5D**). There was no association between EBV viral load in PBMCs and saliva with the presence of a positive T cell response to EBV (**Supplementary Figure 12D**). Furthermore, EBV viral load in PBMCs was not associated with the frequency of T cell subsets (**Supplementary Figure 12E**).

## Discussion

We have shown, in the current study, the pattern of immune cell subsets in healthy individuals across the age span from rural Uganda, as a basis for reference immune values in this, or similar, populations across SSA. Several immune cell subsets varied by age. We have shown that CD4:CD8 T cell ratios of individuals tested, in the current study, were mostly above one and those aged 16-30 years had the highest CD4:CD8 T cell ratio. CD4:CD8 T cell ratio is used to assess immune recovery in immunocompromised individuals (33). Consequently, lower CD4: CD8 T cell ratios have been associated with old age in people living with HIV (34). Since all individuals tested in the current study were HIV-negative and healthy, we anticipated that their CD4:CD8 T cell ratios would be above one as observed.

We have shown a decrease of both naïve CD4+ and CD8+ T cells with increasing age. This has been attributed to thymic involution in adults (35) reducing the number of naïve T cells produced with increasing age. The proportion of naïve CD4 and CD8 T cells we observed was comparable to data from other sub-Saharan African countries (21). However, the proportion of naïve CD4 and CD8 T cells we observed was lower than that observed in age-matched individuals from resource rich countries (13). This difference could be attributed to the high burden of infectious diseases in SSA driving immune ageing. Infection rates of herpesviruses such as CMV, EBV, HHV8, HSV are more common in sub-Saharan Africa (SSA) than elsewhere (5). Furthermore, in SSA, Additionally, herpesvirus infections occur in childhood in SSA as opposed to adolescence in other perts of the world (6–8). Similarly, acute repeated infections such as *P. falciparum* malaria, flu causing viral infections, bacterial infections are very common in SSA (12). These infections accelerate immune aging for example Infection with CMV has been shown to drive immune aging (36) The reduction in naïve T cells is a marker of immune senescence in combination with increased proportions of terminally differentiated T cells (18). Immune senescence is known to increase with increasing age. In the current study, immunosenescent T cells were more frequent in CD8 T cells compared to CD4 T cells. Both CD4+ and CD8+ immunosenescent T cells increased with increasing age, corresponding to the reduction of naïve T cells with age. immunosenescent T cell frequencies in older individuals from the current study were comparable to those reported elsewhere (37). On the other hand, the frequency of immunosenescent T cells amongst younger individuals in the current study were higher than those reported elsewhere (37). The infectious disease burden in SSA including early CMV infections, recurrent *P. falciparum* infections coupled with viral and bacterial infections throught childhood (38) could drive cell replicative senescence in younger adults from SSA. Additionally, activated CD4+ and CD8+ T cells increased with increasing age, implying that immune activation also increases with age.

We observed differences in some cell substes between data analysed using conventional flow cytometry and full spectrum flow cytometry. However, we were not equipped to investigate the differences in cell sub sets by the two flow cytometry methods due to sample limitations but these differences could be partially attributed to the inclusion of the 4-15 years age group in conventional flow cytometry analysis but not the full spectrum flow cytometry.

T cell function, as measured by IFN-γ production by memory EBV-specific T cells, increased with age but reduced in the 55-89 age group. Since infection with EBV in SSA occurs in childhood (39), viral reactivation over the years could have led to the increase in memory EBV-specific T cells with age, while immune senescence may have led to the reduction in older individuals. Additionally, immune-exhausted CD8+ T cells were more frequent in individuals without detectable T cell function, based on this finding, we hypothesise that immune exhaustion plays a role in impairment of immune function to chronic infections such as herpesvirus infections.

As with T cells, B cell production reduces over time, with older individuals having more autoantibodies and less efficient antigen-specific antibodies (40). In the current study, we observed decreasing numbers of CD19+ B cells with increasing age. Previous studies from resource rich countries have reported either decreasing or unchanged B cells with increasing age (41, 42) and no evidence to suggest declining B cell production by the bone marrow with age (43). Furthermore, fewer naïve B cells correlated with increased IgG to EBV VCA, suggesting that either risk factors causing EBV reactivation like infection with *P. falciparum* (31) or infection with EBV reduces the pool of naïve B cells. B cells were classified into three major groups, naïve (IgD+CD27-), memory (IgD-CD27+) and atypical/double negative B cells (IgD-CD27-) (44). The reduction in naïve B cells with age in the current population was compensated by the increase in both atypical/double negative and memory B cells with increasing age. Of these three B cell subsets, memory B cells were the least prevalent. Previous studies, not from SSA have reported an increase in NK cell percentages with age (45, 46). In the current study we report no difference in NK cell subsets with increasing age, although our study may not have been powered to detect significant changes in NK cells.

## Conclusion

We have shown the pattern of immune cell frequencies in an African population across a wide age range including both children and older individuals in addition to younger adults. Major immune cells follow a similar pattern as those reported elsewhere but the frequencies in each age group differ. These differences may be attributed to environmental factors including the higher burden of infections unique to SSA.

## Data availability statement

The original contributions presented in the study are included in the article/**Supplementary Material**. Further inquiries can be directed to the corresponding author.

## Ethics statement

The studies involving humans were approved by Research Institute Research and Ethics Committee (UVRI-REC, reference number: GC/127/16/09/566), the Uganda National Council for Science and Technology (UNCST, reference number: HS2123) and the London School of Hygiene and Tropical Medicine Ethics Committee (reference number: 11881). The studies were conducted in accordance with the local legislation and institutional requirements. Written informed consent for participation in this study was provided by the participants’ legal guardians/next of kin.

## Author contributions

AN: Conceptualization, Methodology, Writing – original draft. MN: Methodology, Writing – review & editing. RRos: Methodology, Writing – review & editing. MK: Methodology, Writing – review & editing. WM: Methodology, Writing – review & editing. DW: Conceptualization, Funding acquisition, Supervision, Writing – review & editing. RN: Funding acquisition, Investigation, Supervision, Writing – review & editing. RRoc: Conceptualization, Funding acquisition, Supervision, Writing – review & editing. SC: Conceptualization, Data curation, Supervision, Writing – review & editing.

## Supplementary material

Supplementary Table 1 Cell subsets and their markers. T_N_: naïve T cells; T_EMRA_: T effector memory RA; T_EM_: effector memory T cells; T_CM_: central memory T cells; B_N_: naïve B cells; B_M_: memory B cells; B_DN_: double (CD27 and IgD) negative B cells.

Supplementary Table 2 Median and IQR of major peripheral blood immune phenotypes in each age group by full spectrum flow cytometry . Median and interquartile range (IQR) computed in STATA version 13. T_N_: naïve T cells; T_EMRA_: T effector memory RA; T_EM_: effector memory T cells; T_CM_: central memory T cells; B_N_: naïve B cells; B_M_: memory B cells; B_DN_: double (CD27 and IgD) negative B cells.

Supplementary Table 3 Median and IQR of major peripheral blood immune phenotypes in each age group by conventional flow cytometry . Median and interquartile range (IQR) computed in STATA version 13. T_N_: naïve T cells; T_EMRA_: T effector memory RA; T_EM_: effector memory T cells; T_CM_: central memory T cells; B_N_: naïve B cells; B_M_: memory B cells; B_DN_: double (CD27 and IgD) negative B cells.

Supplementary Figure 1 gating strategy of conventional flow cytometry data CD4+, CD8+ T cells and B cells, were gated using flowJo 10.8.1 software following acquisition on an LSR-2 flow cytometer of three different panels. N: naive, CM: central memory, EM: effector memory, TEMRA: terminally differentiated effector memory. TD: terminally differentiated, PBMCS: peripheral blood mononuclear cells.

Supplementary Figure 2 CD4+ T cell subsets measured using full spectrum flow cytometry by age. Cell subsets were gated using flowJo 10.8.1 software following acquisition on a 5 laser Cytek Aurora cytometer. CM: central memory, EM: effector memory, TEMRA: T effector memory RA. R2 and P values obtained using linear regression.

Supplementary Figure 3 CD4+ T cell subsets measured using conventional flow cytometry by age. Cell subsets were gated using flowJo 10.8.1 software following acquisition on an LSR-II flow cytometer. CM: central memory, EM: effector memory, TEMRA: T effector memory RA. R2 and P values obtained using linear regression.

Supplementary Figure 4 CD8+ T cell subsets measured using full spectrum flow cytometry by age. Cell subsets were gated using flowJo 10.8.1 software following acquisition on a 5 laser Cytek Aurora cytometer. CM: central memory, EM: effector memory, TEMRA: T effector memory RA. R2 and P values obtained using linear regression.

Supplementary Figure 5 CD8+ T cell subsets measured using conventional flow cytometry by age. Cell subsets were gated using flowJo 10.8.1 software following acquisition on an LSR-II flow cytometer. CM: central memory, EM: effector memory, TEMRA: T effector memory RA. R2 and P values obtained using linear regression.

Supplementary Figure 6 B cell subsets measured using full spectrum flow cytometry by age. Cell subsets were gated using flowJo 10.8.1 software following acquisition on a 5 laser Cytek Aurora cytometer. DN: double (CD27 & IgD) negative.

Supplementary Figure 7 B cell subsets measured using conventional flow cytometry by age. Cell subsets were gated using flowJo 10.8.1 software following acquisition on an LSR-II flow cytometer. DN: double (CD27 & IgD) negative.

Supplementary Figure 8 B cell subsets measured using conventional flow cytometry by age. Cell subsets were gated using flowJo 10.8.1 software following acquisition on an LSR-II flow cytometer. DN: double (CD27 & IgD) negative.

Supplementary Figure 9 NK cells and monocytes subsets measured using full spectrum flow cytometry by age. Cell subsets were gated using flowJo 10.8.1 software following acquisition on a 5 laser Cytek Aurora cytometer.

Supplementary Figure 10 Percentage frequency of CD4+ T cells, CD8+ T cells, B cells NK cells and monocytes subsets in males and females. CD4+ T cells, CD8+ T cell, B cells, NK cells and monocytes were gated using flowJo 10.8.1 software following acquisition on a 5 laser Cytek Aurora cytometer. DN: double negative, CM: central memory, EM: effector memory, TEMRA: terminally differentiated effector memory.

Supplementary Figure 11 Percentage frequency of CD4+ T cells, CD8+ T cells and B cells subsets in males and females.CD4+ T cells, CD8+ T cell and B cells were gated using flowJo 10.8.1 software following acquisition on an LSR-II flow cytometer using three antibody panels. DN: double negative/atypical, CM: central memory, EM: effector memory, TEMRA: terminally differentiated effector memory.

Supplementary Figure 12 Correlation between B cell subsets and IgG responses to EBV (Epstein-Barr virus) viral capsid antigen-VCA **(A, B)**. The percentages of T cells subsets in individuals with and without an IFN-γ responses to EBV **(C)**; EBV viral load in individuals with and without a positive T cell response to EBV **(D)**, T cell subsets in individuals with and without EBV virus in peripheral blood mononucleaf cells-PBMC **(E)**. IFN-γ responses to EBV (Epstein-Barr virus) peptide pool were measured using enzyme linked immunosorbent spot (ELISpot) assay; IgG to EBV-VCA (viral capsid antigen) was quantified using multiplex bead assay. Statistical analysis methods used include linear regression **(A, B)**, Wilcoxon Rank Sum Test **(C–E)**.
