# Supplementary material for: Immune cell phenotype and function patterns across the life course in individuals from rural Uganda
Source: Front Immunol. 2024 Mar 18;15:1356635. doi: 10.3389/fimmu.2024.1356635 (PMC10982424; doi:10.3389/fimmu.2024.1356635)
Supplement: Supplementary Table 1 — Cell subsets and their markers. TN: naïve T cells; TEMRA: T effector memory RA; TEM: effector memory T cells; TCM: central memory T cells; BN: naïve B cells; BM: memory B cells; BDN: double (CD27 and IgD) negative B cells. [file Table_1.pdf]

Supplementary Table 1: Cell subsets and their markers

number	phenotype	Cell markers
1	CD4+	CD3+ CD4+
2	CD4+ T_N	CD3+ CD4+ CCR7+ CD45RA+
3	CD4+ T_{EMRA}	CD3+ CD4+ CCR7- CD45RA+
4	CD4+ T_{EM}	CD3+ CD4+ CCR7- CD45RA-
5	CD4+ T_{CM}	CD3+ CD4+ CCR7+ CD45RA-
6	CD4+ CD57+ KLRG1+	CD3+ CD4+ CD28- CD27- CD57+ KLRG1+
7	CD4+ CD38+ HLADR+	CD3+ CD4+ CD38+ HLADR+
8	CD4+ PD1+	CD3+ CD4+ PD1+
9	CD4+ LAG3+	CD3+ CD4+ LAG3+
10	CD8+	CD3+ CD8+
11	CD8+ T_N	CD3+ CD8+ CCR7+ CD45RA+
12	CD8+ T_{EMRA}	CD3+ CD8+ CCR7- CD45RA+
13	CD8+ T_{EM}	CD3+ CD8+ CCR7- CD45RA-
14	CD8+ T_{CM}	CD3+ CD8+ CCR7+ CD45RA-
15	CD8+ CD57+ KLRG1+	CD3+ CD8+ CD28- CD27- CD57+ KLRG1+
16	CD8+ CD38+ HLADR+	CD3+ CD8+ CD38+ HLADR+
17	CD8+ PD1+	CD3+ CD8+ PD1+
18	CD8+ LAG3+	CD3+ CD8+ LAG3+
18	CD19+	CD19+
20	B_N	CD19+ CD10- CD27- IgD+
21	B_M	CD19+ CD10- CD27+ IgD-
22	B_{DN}	CD19+ CD10- CD27- IgD-
23	B_{DN1}	CD19+ CD10- CD27- IgD- CD38+ CD21+
24	B_{DN2}	CD19+ CD10- CD27- IgD- CD38- CD21-
25	B_N CD38+ CD21+	CD19+ CD10- CD27- IgD+ CD38+ CD21+
26	B_N CD38- CD21-	CD19+ CD10- CD27- IgD+ CD38- CD21-
27	B_M CD38+ CD21+	CD19+ CD10- CD27+ IgD- CD38+ CD21+
28	B_M CD38- CD21-	CD19+ CD10- CD27+ IgD- CD38- CD21-
29	CD56- NK cells	CD3-CD19-CD14- CD16++ CD56-
30	CD56dim NK cells	CD3-CD19-CD14- CD16+/- CD56+
31	CD56 bright NK cells	CD3-CD19-CD14- CD16- CD56++
32	NKG2D+ NK cells	CD3-CD19-CD14- CD56+ NKG2D+
33	NKG2A+ NK cells	CD3-CD19-CD14- CD56+ NKG2A+
34	Classical monocytes	CD3-CD19-CD56- CD16- CD14+
35	Intermediate monocytes	CD3-CD19-CD56- CD16+ CD14+
36	Non-classical monocytes	CD3-CD19-CD56- CD16+ CD14-

T_N: naïve T cells; T_{EMRA}: T effector memory RA; T_{EM}: effector memory T cells; T_{CM}: central memory T cells; B_N: naïve B cells; B_M: memory B cells; B_{DN}: double (CD27 and IgD) negative B cells
