# Supplementary material for: Immune cell phenotype and function patterns across the life course in individuals from rural Uganda
Source: Front Immunol. 2024 Mar 18;15:1356635. doi: 10.3389/fimmu.2024.1356635 (PMC10982424; doi:10.3389/fimmu.2024.1356635)
Supplement: Supplementary Table 2 — Median and IQR of major peripheral blood immune phenotypes in each age group by full spectrum flow cytometry . Median and interquartile range (IQR) computed in STATA version 13. TN: naïve T cells; TEMRA: T effector memory RA; TEM: effector memory T cells; TCM: central memory T cells; BN: naïve B cells; BM: memory B cells; BDN: double (CD27 and IgD) negative B cells. [file Table_2.pdf]

Supplementary Table 2: Median and IQR of major peripheral blood immune phenotypes in each age group by full spectrum flow cytometry

number	phenotype	16-30 years median (IQR)	31-55 years median (IQR)	56-89 years median (IQR)
1	CD4+	54.4 (47.9- 58.7)	51.3 (41.1- 58.4)	57 (48.8 – 62.3)
2	CD4+ T_N	34.4 (31.7 – 41.8)	25.45 (17 – 29.5)	21 (12-29.2)
3	CD4+ T_{EMRA}	5.55 (4.27 – 7.53)	4.83 (3.98 – 7.43)	5.47 (3.7 4– 7.69)
4	CD4+ T_{EM}	33.3 (28.3 – 38.5)	36 (25.3 – 40.4)	37.6 (31.8 – 44.9)
5	CD4+ T_{CM}	24.1 (20 – 27.1)	29.65 (23.9 – 36.9)	31.5 (25.8 – 35.4)
6	CD4+ CD57+ KLRG1+	1.67 (0.96- 2.93)	2.62 (1.51- 5.49)	5.92 (2.2 – 8.23)
7	CD4+ CD38+ HLADR+	6.8 (4.17 – 8.25)	5.62 (3.58 – 10.9)	5.19 (3.49- 8.87)
8	CD4+ PD1+	16.02 (13.28- 18.13)	16.75 (11.62 – 19.53)	19.19 (16.46 – 24.86)
9	CD4+ LAG3+	2.78 (2.26 – 4.69)	3.05 (1.87 – 4.36)	3.45 (2.5 – 4.74)
10	CD8+	31.7 (29 – 35.2)	35.55 (28.7 - 41.9)	30.7 (25- 41.1)
11	CD8+ T_N	26.5 (23.9 – 34.3)	16.05 (10.5 – 30.1)	7.22 (4.86 – 18.7)
12	CD8+ T_{EMRA}	51.8 (43.4 – 58.2)	50.4 (43.2 – 61.3)	50.5 (44.3 – 66.9)
13	CD8+ T_{EM}	13.4 (11.2 – 19.4)	24.2 (13.8 – 31.2)	26.3 (21.9 – 39.8)
14	CD8+ T_{CM}	1.3 (0.88 – 1.98)	2.14 (1.41 – 2.8)	2.69 (1.76 – 3.75)
15	CD8+ CD57+ KLRG1+	27.7 (21.2 – 36.7)	28.8 (18.8 – 43.2)	32.8 (23.3 – 38.9)
16	CD8+ CD38+ HLADR+	8.74 (4.65 – 12.4)	7.57 (5.06 – 13.6)	8.43 (4.9 – 14.8)
17	CD8+ PD1+	10.8 (5.53 – 12.03)	8.0 (6.55 – 10.8)	9.24 (5.44 – 14.13)
18	CD8+ LAG3+	1.39 (1.2 – 2.29)	1.73 (1.23 – 2.73)	2.4 (1.57 – 2.99)
18	CD19+	9.45 (7.91 – 11.6)	9.69 (6.52 – 12.9)	8.26 (6.18 – 11.8)
20	B_N	30.4 (22.9 – 45.5)	16.6 (12.3 – 24.6)	12 (8.57 – 23.9)
21	B_M	27.3 (15.5 – 41.4)	38.65 (24.5 – 53.9)	31.1 (21.8 – 46.8)
22	B_{DN}	30.9 (18.3 – 43.8)	27.35 (15 – 45.3)	35.8 (28.3 – 64.2)
23	B_{DN1}	8.32 (5.6 – 9.56)	4.95 (3.04 – 7.8)	4.36 (2.81 – 8.36)
24	B_{DN2}	7.89 (3.34 – 17.4)	5.61 (2.52 – 19.2)	16.1 (7.97 -22.2)
25	B_N CD38+ CD21+	11.9 (7.78 – 15.7)	5.62 (2.47 – 12.3)	3.98 (1.67 – 11)
26	B_N CD38- CD21-	3.26 (2.03 – 5.43)	1.3 (0.79 -4)	1.68 (0.81 – 3.97)
27	B_M CD38+ CD21+	6.28 (3.32 – 10.1)	5.33 (3.82 – 10.6)	3.57 (1.97 -5.47)
28	B_M CD38- CD21-	7.6 (3.59 – 12.5)	13.75 (7.85 – 23.6)	14.7 (7.4 – 20.7)
29	CD56- NK cells	15 (8.85 – 21)	19.8 (12.9 – 25.4)	16.6 (13.7 – 29.1)
30	CD56dim NK cells	26.9 (10.5 – 33.1)	22.5 (14.1 – 35.6)	27.2 (17.9 – 35.9)
31	CD56 bright NK cells	1.88 (0.73 – 3.16)	1.85 (1.14 – 2.21)	1.09 (0.58 – 2.16)

32	NKG2D+ NK cells	14.55 (4.99 – 17.62)	11.14 (6.39 – 19.97)	10.16 (6.53 – 13.9)
33	NKG2A+ NK cells	5.2 (1.14 – 6.91)	3.80 (0.91 – 7.01)	1.36 (0.78 – 2.77)
34	Classical monocytes	44.4 (24.9 – 53.2)	45.25 (36.1 – 61)	40.8 (28.1 – 50.5)
35	Intermediate monocytes	3.15 (0.97 – 3.81)	4.51 (1.68 – 6.55)	1.76 (0.65 – 5.01)
36	Non-classical monocytes	8.37 (4.75 – 13.1)	8.77 (5.02 – 11.9)	11.7 (7.48 – 14.6)

Median and interquartile range (IQR) computed in STATA version 13. T_N: naïve T cells; T_{EMRA}: T effector memory RA; T_{EM}: effector memory T cells; T_{CM}: central memory T cells; B_N: naïve B cells; B_M: memory B cells; B_{DN}: double (CD27 and IgD) negative B cells
