# Supplementary material for: Immune cell phenotype and function patterns across the life course in individuals from rural Uganda
Source: Front Immunol. 2024 Mar 18;15:1356635. doi: 10.3389/fimmu.2024.1356635 (PMC10982424; doi:10.3389/fimmu.2024.1356635)
Supplement: Supplementary Table 3 — Median and IQR of major peripheral blood immune phenotypes in each age group by conventional flow cytometry . Median and interquartile range (IQR) computed in STATA version 13. TN: naïve T cells; TEMRA: T effector memory RA; TEM: effector memory T cells; TCM: central memory T cells; BN: naïve B cells; BM: memory B cells; BDN: double (CD27 and IgD) negative B cells. [file Table_3.pdf]

Supplementary Table 3: Median and IQR of major peripheral blood immune phenotypes in each age group by conventional flow cytometry

number	phenotype	4-15 years median (IQR)	16-30 years median (IQR)	31-55 years median (IQR)	56-89 years median (IQR)
1	CD4+	66.45 (61-69.7)	66.5 (61.75 – 71.5)	62.3 (57.5 – 71.4)	65.9 (56.3 – 67.1)
2	CD4+ T_N	45.7 (40.9 – 54)	28.2 (25.15 – 36.65)	30 (18.2 – 33.9)	26.1 (15.2 – 31.9)
3	CD4+ T_{EMRA}	9.18 (6.16 – 12.7)	9.81 (5.96 - 11.85)	6.46 (4.66 – 8.87)	9.17 (5.47 – 11.2)
4	CD4+ T_{EM}	15.45 (12.3 – 23.2)	26.75 (21.15 – 30.75)	25.1 (18.5 – 37.5)	23.7 (15.5 – 36.2)
5	CD4+ T_{CM}	23.55 (20.4 – 26.8)	33.45 (30.2 – 37.35)	40.6 (34.2 – 46.6)	39.2 (33.7 – 49.8)
6	CD4+ CD57+	0.24 (0.14-0.71)	1.02 (0.42 – 1.96)	2.04 (0.66 – 3.54)	2.28 (1.51 – 4.68)
7	CD4+ HLADR+	0.27 (0.21 – 0.34)	0.32 (0.24 – 0.38)	0.46 (0.36 – 0.52)	0.84 (0.67 - 1.14)
8	CD4+ PD1+	0.24 (0.16 – 0.29)	0.20 (0.04 – 0.35)	0.07 (0.01 – 0.22)	0.21 (0.11 – 0.27)
9	CD8+	21.1 (19.1 – 24.2)	19.7 (17.2 – 25.65)	23.4 (19.2 – 30.7)	26.8 (23.7 – 30.4)
10	CD8+ T_N	38.6 (30.8 – 44.6)	33.5 (23 – 38.45)	27.6 (21.5 – 31.7)	29.8 (24 – 30.8)
11	CD8+ T_{EMRA}	12.3 (8.54 – 19.4)	10.9 (8.35 – 14.95)	9.06 (6.02 – 14.9)	6.81 (6.01 – 9.67)
12	CD8+ T_{EM}	13.55 (11.1 – 18.2)	14.15 (12.65 – 20.95)	13.8 (10.3 – 16.3)	10.4 (8.63 – 14.2)
13	CD8+ T_{CM}	32.2 (22.4 – 39.9)	39.45 (33.3 – 46.55)	46.8 (35.9 – 53.4)	50.7 (43.8 – 56)
14	CD8+ CD57+	23.65 (16.9 – 28)	27.2 (19.1 – 36.15)	33.6 (16.7 – 40.1)	39.9 (26.9 – 55.6)
15	CD8+ HLADR+	0.53 (0.41 – 0.78)	0.61 (0.48 - 0.91)	0.64 (0.50 – 0.87)	1.13 (0.81 – 1.48)
16	CD8+ PD1+	0.23 (0.15 – 0.28)	0.29 (0.07 – 0.36)	0.05 (0.01 – 0.24)	0.15 (0.06 – 0.22)
17	CD19+	13.9 (10.8 – 16.8)	10.9 (7.28 – 13)	9.86 (7.15 – 12.8)	8.46 (5.44 – 10.9)
18	B_N	39.2 (35.5 – 42.4)	37.4 (31.6 – 41.2)	32 (23.4 – 38.8)	27.15 (19.6 – 33.8)
18	B_M	10.3 (7.55 – 13.4)	17.5 (9.39 – 19.9)	17.7 (12.6 – 20.1)	12.9 (9.38 – 21.85)
20	B_{DN}	16.1 (14.6 – 22)	22.5 (18.5 – 33.1)	32.15 (23.9 - 42.9)	39.7 (32.25 – 44.6)
21	B_{DN1}	5.73 (4.77 – 6.36)	5.57 (3.43 – 9.71)	5.92 (3.55 – 8.31)	3.72 (2.97 – 5.41)
22	B_{DN2}	5.13 (3.05 – 7.18)	8.74 (4.68 – 15.7)	14.8 (8.84-17.7)	21.45 (15.7 – 26.55)
23	B_{DN} IgG+	6.83 (3.96 – 8.23)	12.2 (7.44 – 13.7)	11.25 (6.87 – 14)	7.94 (5.77 – 12.05)
24	B_{DN} IgM+	3.70 (1.97 – 8.19)	2.35 (1.48 – 6.52)	5.41 (2.87-8.78)	11.9 (5.5 – 17.9)

25	B_N CD38+ CD21+	25.7 (23.8 – 29)	25.6 (13.4 - 31.2)	24 (11.3 – 27.4)	12.95 (4.94 – 15.45)
26	B_N CD38- CD21-	2.31 (1.76 – 3.82)	2.24 (1.25 – 4.28)	2.94 (2.18 – 3.76)	5.10 (4.14 - 8.55)
27	B_M CD38+ CD21+	4.83 (2.57 - 5.75)	5.62 (4.07 – 7.92)	6.30 (4.67 – 8.02)	3.73 (1.99 – 6.84)
28	B_M CD38- CD21-	1.01 (0.78 – 1.47)	1.72 (0.74 – 2.88)	2.73 (1.54 – 4.17)	3.37 (1.72 – 4.1)
29	B_M IgG+	3.42 (1.73 – 4.97)	5.46 (3.9 – 7.99)	6.03 (4.74 – 7.83)	3.59 (2.38 – 5.51)
30	B_M IgM+	1.32 (1.05 – 1.86)	0.89 (0.54 – 1.89)	1.76 (1.18 – 2.37)	1.89 (1.31 – 2.39)

Median and interquartile range (IQR) computed in STATA version 13. T_N: naïve T cells; T_{EMRA}: T effector memory RA; T_{EM}: effector memory T cells; T_{CM}: central memory T cells; B_N: naïve B cells; B_M: memory B cells; B_{DN}: double (CD27 and IgD) negative B cells
