# Supplementary material for: Immune cell phenotype and function patterns across the life course in individuals from rural Uganda
Source: Front Immunol. 2024 Mar 18;15:1356635. doi: 10.3389/fimmu.2024.1356635 (PMC10982424; doi:10.3389/fimmu.2024.1356635)
Supplement: Supplementary Figure 1 — gating strategy of conventional flow cytometry data CD4+, CD8+ T cells and B cells, were gated using flowJo 10.8.1 software following acquisition on an LSR-2 flow cytometer of three different panels. N: naive, CM: central memory, EM: effector memory, TEMRA: terminally differentiated effector memory. TD: terminally differentiated, PBMCS: peripheral blood mononuclear cells. [file Image_1.pdf]

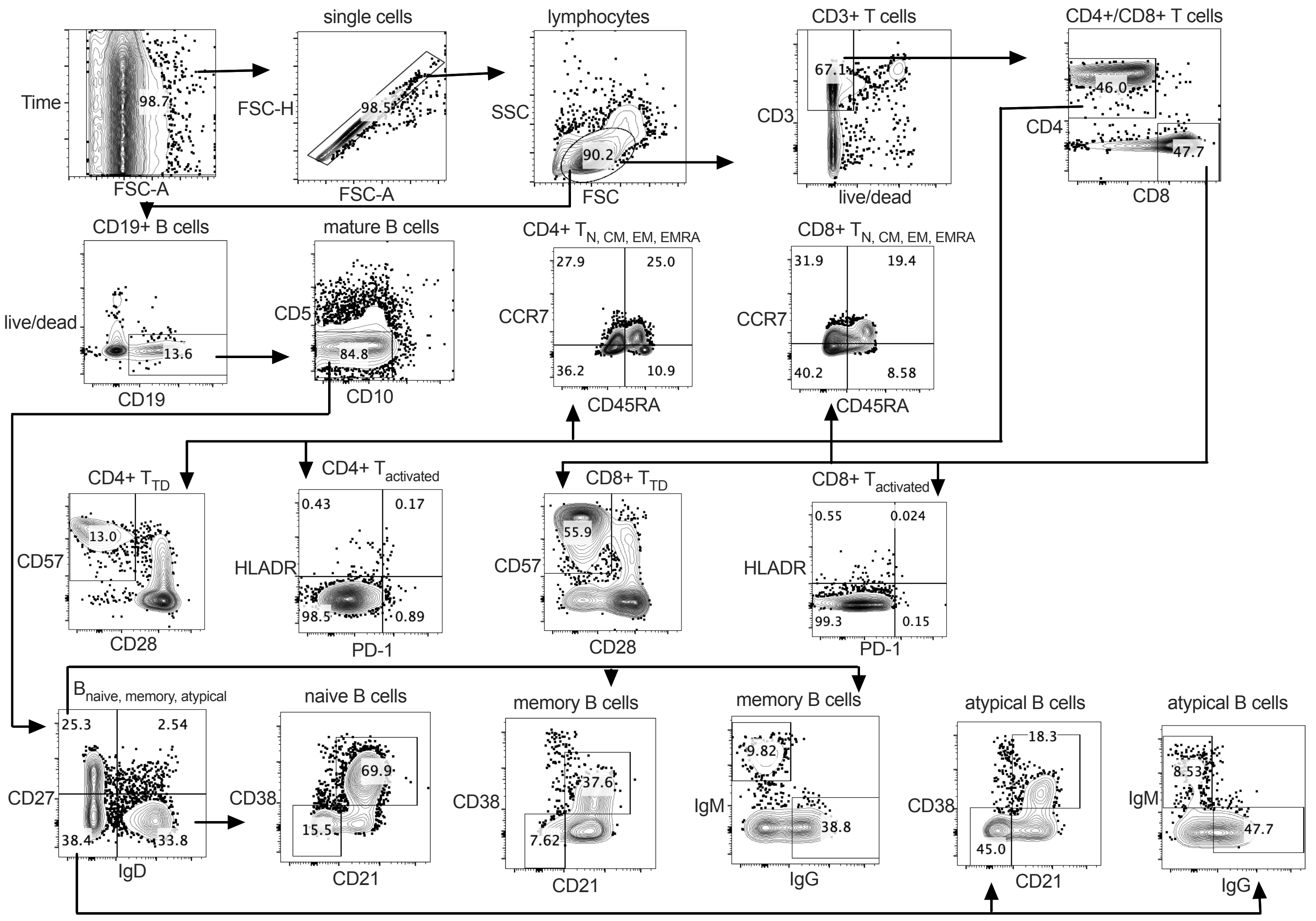

Supplementary Figure 1: gating strategy of conventional flow cytometry data CD4+, CD8+ T cells and B cells, were gated using flowJo 10.8.1 software following acquisition on an LSR-2 flow cytometer of three different panels. N: naive, CM: central memory, EM: effector memory, T_{EMRA}: T effector memory RA. TD: terminally differentiated, PBMCs: peripheral blood mononuclear cells.
