# Supplementary material for: Immune cell phenotype and function patterns across the life course in individuals from rural Uganda
Source: Front Immunol. 2024 Mar 18;15:1356635. doi: 10.3389/fimmu.2024.1356635 (PMC10982424; doi:10.3389/fimmu.2024.1356635)
Supplement: Supplementary Figure 4 — CD8+ T cell subsets measured using full spectrum flow cytometry by age. Cell subsets were gated using flowJo 10.8.1 software following acquisition on a 5 laser Cytek Aurora cytometer. CM: central memory, EM: effector memory, TEMRA: T effector memory RA. R2 and P values obtained using linear regression. [file Image_4.pdf]

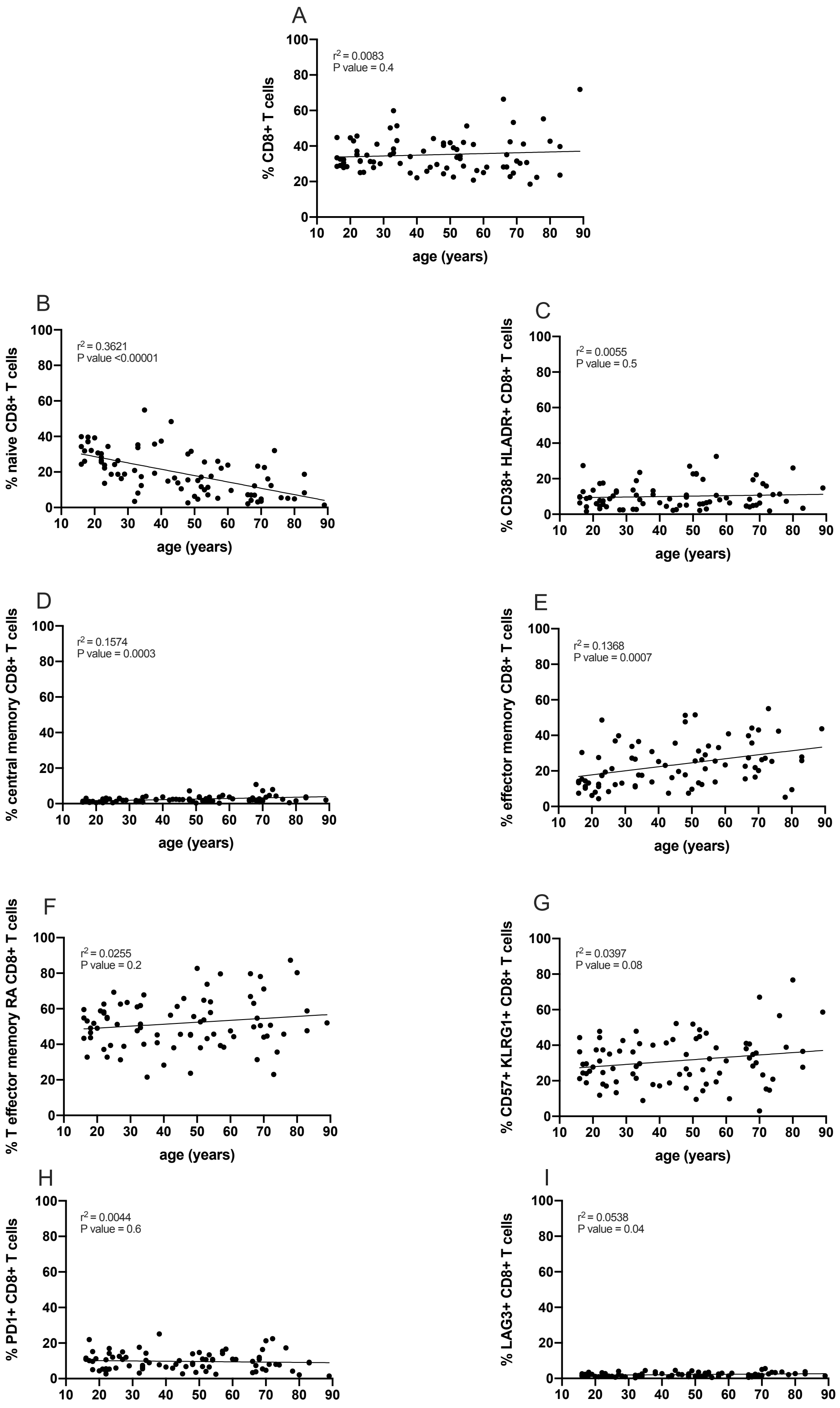

Supplementary Figure 4: CD8+ T cell subsets measured using full spectrum flow cytometry by age. Cell subsets were gated using flowJo 10.8.1 software following acquisition on a 5 laser Cytek Aurora cytometer. CM: central memory, EM: effector memory, TEMRA: T effector memory RA. R2 and P values obtained using linear regression.
