# Supplementary material for: Immune cell phenotype and function patterns across the life course in individuals from rural Uganda
Source: Front Immunol. 2024 Mar 18;15:1356635. doi: 10.3389/fimmu.2024.1356635 (PMC10982424; doi:10.3389/fimmu.2024.1356635)
Supplement: Supplementary Figure 6 — B cell subsets measured using full spectrum flow cytometry by age. Cell subsets were gated using flowJo 10.8.1 software following acquisition on a 5 laser Cytek Aurora cytometer. DN: double (CD27 & IgD) negative. [file Image_6.pdf]

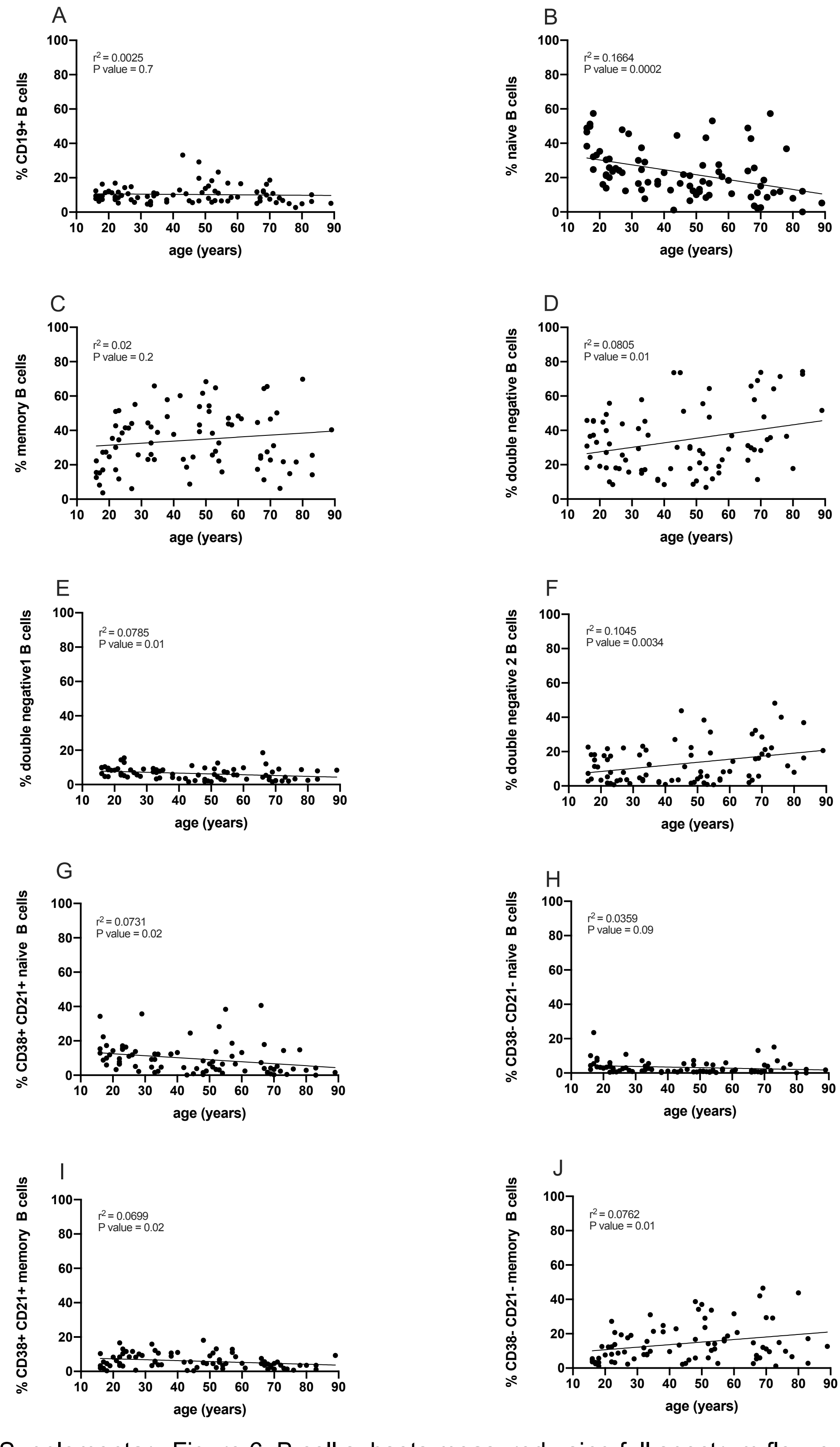

Supplementary Figure 6: B cell subsets measured using full spectrum flow cytometry by age. Cell subsets were gated using flowJo 10.8.1 software following acquisition on a 5 laser Cytek Aurora cytometer. DN: double (CD27 & IgD) negative.
