# Supplementary material for: Immune cell phenotype and function patterns across the life course in individuals from rural Uganda
Source: Front Immunol. 2024 Mar 18;15:1356635. doi: 10.3389/fimmu.2024.1356635 (PMC10982424; doi:10.3389/fimmu.2024.1356635)
Supplement: Supplementary Figure 10 — Percentage frequency of CD4+ T cells, CD8+ T cells, B cells NK cells and monocytes subsets in males and females. CD4+ T cells, CD8+ T cell, B cells, NK cells and monocytes were gated using flowJo 10.8.1 software following acquisition on a 5 laser Cytek Aurora cytometer. DN: double negative, CM: central memory, EM: effector memory, TEMRA: terminally differentiated effector memory. [file Image_10.pdf]

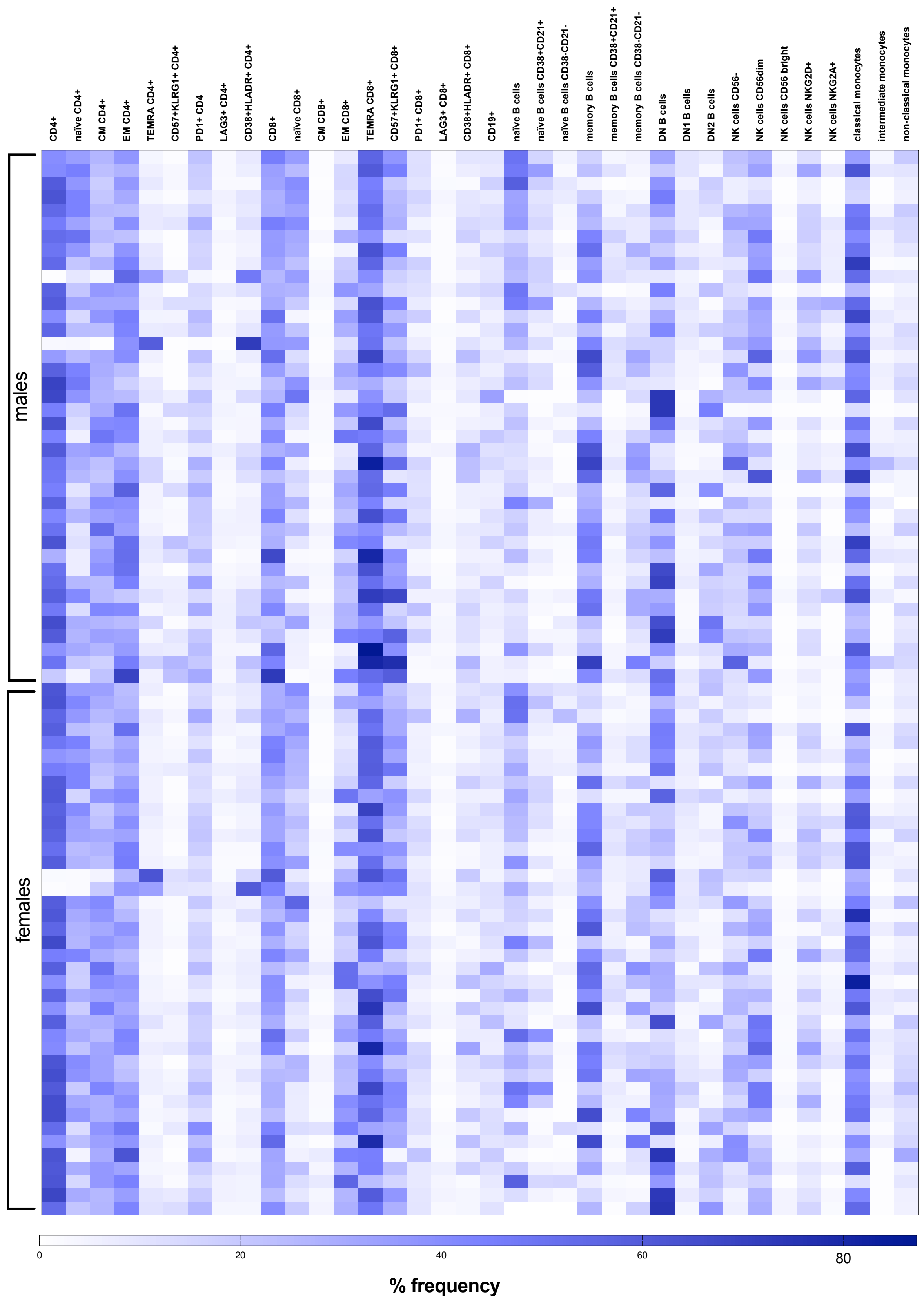

Supplementary Figure 10: Percentage frequency of CD4+ T cells, CD8+ T cells, B cells, NK cells and monocytes subsets in males and females. CD4+ T cells, CD8+ T cell , B cells, NK cells and monocytes were gated using flowJo 10.8.1 software following acquisition on a 5 laser Cytek Aurora cytometer. DN: double negative, CM: central memory, EM: effector memory, TEMRA: terminally differentiated effector memory.
