# Supplementary material for: Immune cell phenotype and function patterns across the life course in individuals from rural Uganda
Source: Front Immunol. 2024 Mar 18;15:1356635. doi: 10.3389/fimmu.2024.1356635 (PMC10982424; doi:10.3389/fimmu.2024.1356635)
Supplement: Supplementary Figure 11 — Percentage frequency of CD4+ T cells, CD8+ T cells and B cells subsets in males and females.CD4+ T cells, CD8+ T cell and B cells were gated using flowJo 10.8.1 software following acquisition on an LSR-II flow cytometer using three antibody panels. DN: double negative/atypical, CM: central memory, EM: effector memory, TEMRA: terminally differentiated effector memory. [file Image_11.pdf]

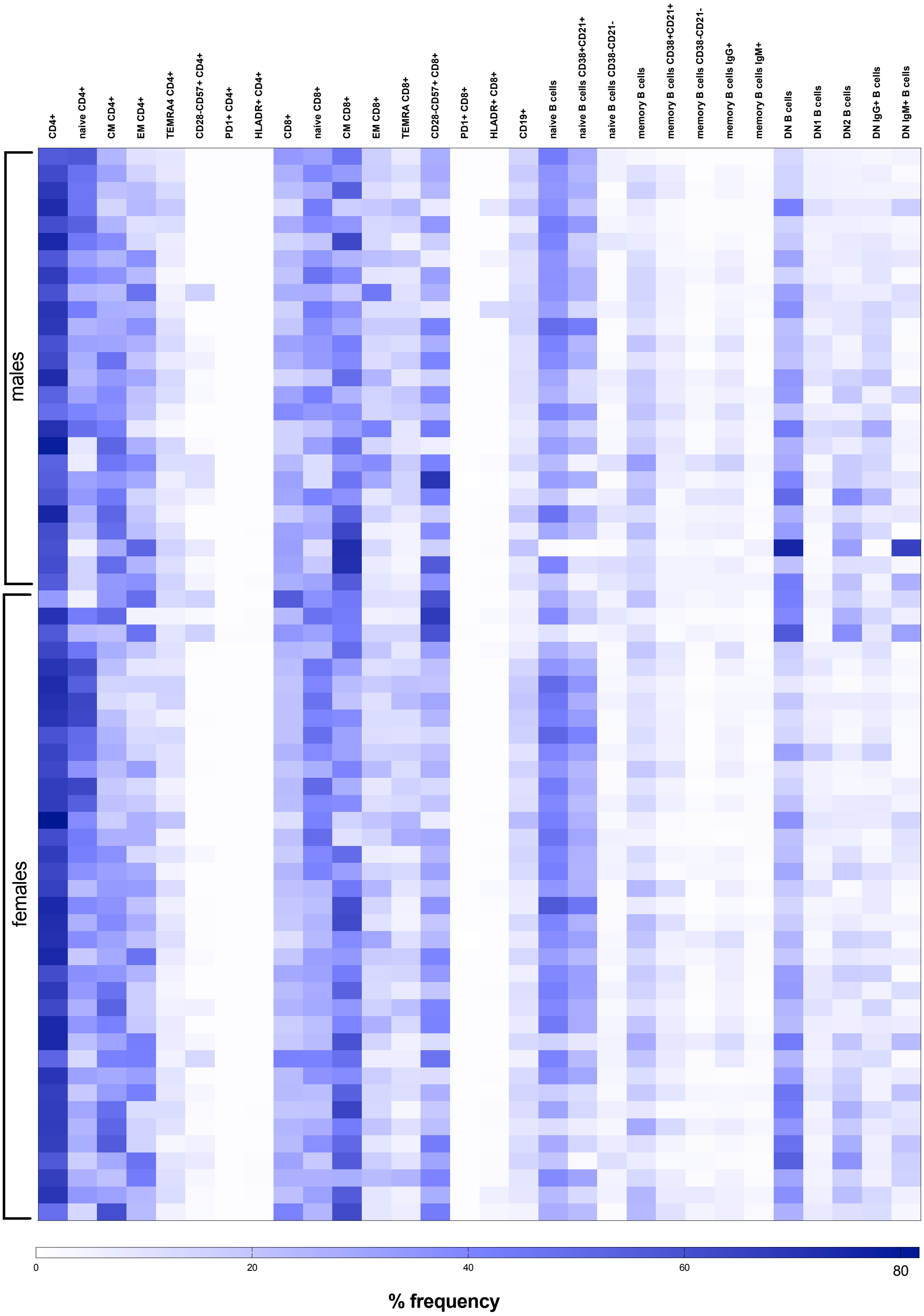

Supplementary Figure 11: Percentage frequency of CD4+ T cells, CD8+ T cells and B cells subsets in males and females.CD4+ T cells, CD8+ T cell and B cells were gated using flowJo 10.8.1 software following acquisition on an LSR-II flow cytometer using three antibody panels. DN: double negative/atypical, CM: central memory, EM: effector memory, TEMRA: terminally differentiated effector memory.
