# Supplementary material for: Immune cell phenotype and function patterns across the life course in individuals from rural Uganda
Source: Front Immunol. 2024 Mar 18;15:1356635. doi: 10.3389/fimmu.2024.1356635 (PMC10982424; doi:10.3389/fimmu.2024.1356635)
Supplement: Supplementary Figure 12 — Correlation between B cell subsets and IgG responses to EBV (Epstein-Barr virus) viral capsid antigen-VCA (A, B). The percentages of T cells subsets in individuals with and without an IFN-γ responses to EBV (C); EBV viral load in individuals with and without a positive T cell response to EBV (D), T cell subsets in individuals with and without EBV virus in peripheral blood mononucleaf cells-PBMC (E). IFN-γ responses to EBV (Epstein-Barr virus) peptide pool were measured using enzyme linked immunosorbent spot (ELISpot) assay; IgG to EBV-VCA (viral capsid antigen) was quantified using multiplex bead assay. Statistical analysis methods used include linear regression (A, B), Wilcoxon Rank Sum Test (C–E). [file Image_12.pdf]

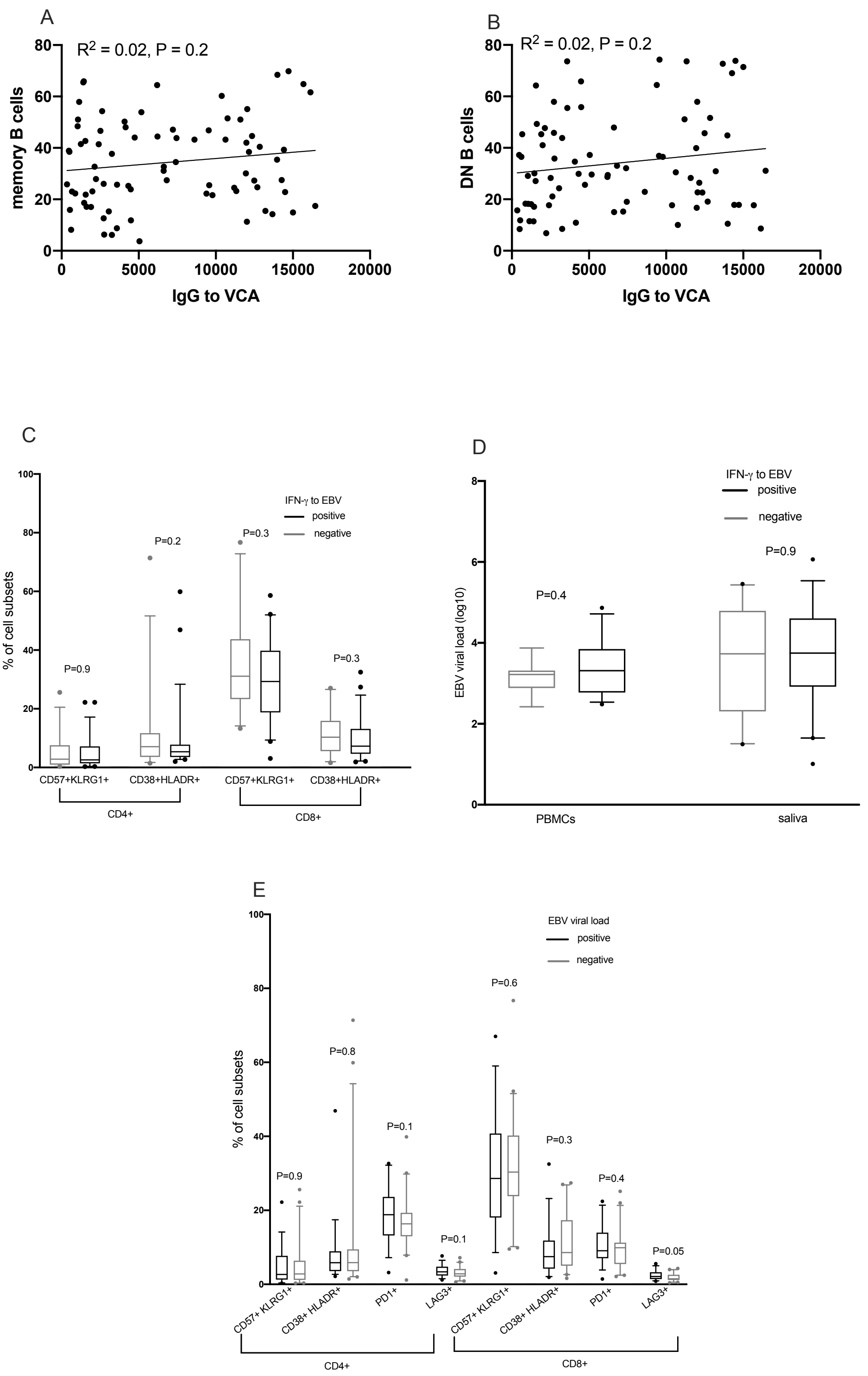

Supplementary Figure 12: Correlation between B cell subsets and IgG responses to EBV (Epstein-Barr virus) viral capsid antigen-VCA (A & B). The percentages of T cells subsets in individuals with and without an IFN-γ responses to EBV (C); EBV viral load in individuals with and without a positive T cell response to EBV (D), T cell subsets in individuals with and without EBV virus in peripheral blood mononuclear cells-PBMC (E). IFN-γ responses to EBV (Epstein-Barr virus) peptide pool were measured using enzyme linked immunosorbent spot (ELISpot) assay; IgG to EBV-VCA (viral capsid antigen) was quantified using multiplex bead assay. Statistical analysis methods used include linear regression (A & B), Wilcoxon Rank Sum Test (C, D, E).
